# Diagnostic Performance of a Novel *CXCL10* mRNA Release Assay for *Mycobacterium tuberculosis* Infection

**DOI:** 10.3389/fmicb.2022.825413

**Published:** 2022-03-30

**Authors:** Liping Pan, Mailing Huang, Hongyan Jia, Guofang Deng, Yu Chen, Rongrong Wei, Mingxia Zhang, Xin Li, Qi Sun, Mutong Fang, Pengfei Ren, Aiying Xing, Qi Chen, Xinxin Li, Boping Du, Tao Chen, Mengqiu Gao, Zongde Zhang

**Affiliations:** ^1^Beijing Chest Hospital, Beijing Key Laboratory for Drug Resistant Tuberculosis Research, Beijing Tuberculosis and Thoracic Tumor Research Institute, Capital Medical University, Beijing, China; ^2^Department of Tuberculosis, Beijing Chest Hospital, Beijing Tuberculosis and Thoracic Tumor Research Institute, Capital Medical University, Beijing, China; ^3^Department of Pulmonary Medicine, The Third People’s Hospital of Shenzhen, Shenzhen, China; ^4^Department of Tuberculosis, Henan Provincial Infectious Disease Hospital, Zhengzhou, China; ^5^Laboratory Medical Center, The Third People’s Hospital of Shenzhen, Guangdong Key Lab of Emerging Infectious Diseases, Shenzhen, China; ^6^Laboratory Medical Center, Henan Provincial Infectious Disease Hospital, Zhengzhou, China

**Keywords:** tuberculosis, *M.tb* infection, molecular diagnosis, mRNA, T-SPOT.TB, *CXCL10*

## Abstract

One-fourth of the world’s population has been infected with *Mycobacterium tuberculosis* (*M.tb*). Although interferon-gamma release assays (IGRAs) have been shown to be valid methods for identifying *M.tb* infection and auxiliary methods for diagnosis of active tuberculosis (TB), lower sensitivity and higher indeterminate rate were often detected among immunosuppressed patients. IP-10 was an alternative biomarker due to the higher expression level after *M.tb* antigen stimulation, but whether *CXCL10* mRNA (the gene that transcribes for the IP-10 protein) can be used as a target for *M.tb* infection diagnosis was limited. Therefore, we aimed to evaluate the performance of a novel *M.tb*-specific *CXCL10* mRNA release assay in diagnosis of *M.tb* infection. Suspected TB patients and healthy controls were prospectively recruited between March 2018 and November 2019 from three hospitals in China. *CXCL10* mRNA release assay and traditional interferon-gamma release assay (T-SPOT.TB) were simultaneously performed on peripheral blood. Of the 1,479 participants enrolled in the study, 352 patients with definite TB and 153 healthy controls were analyzed. *CXCL10* mRNA release assay provided a sensitivity of 93.9% (95% CI = 90.8–96.2%) and a specificity of 98.0% (95% CI = 94.3–99.6%) in the diagnosis of *M.tb* infection, respectively, while T-SPOT.TB gave a sensitivity of 94.5% (95% CI = 91.5–96.6%) and a specificity of 100% (95% CI = 97.6–100.0%) in the diagnosis of *M.tb* infection, respectively. The diagnostic performance of *CXCL10* mRNA release assay was consistent with T-SPOT.TB, with a total coincidence rate of 95.0% (95% CI = 93.0–96.9%) and a Cohen’s kappa value of 0.89 (0.84–0.93, *p* < 0.001). However, among TB patients with HIV co-infection (*n* = 14), *CXCL10* mRNA release assay presented significantly higher positive rate [92.9% (66.1–99.8%) *vs*. 61.5% (31.6–86.1%), *p* = 0.029] than those of T-SPOT.TB. These results suggested that *M.tb*-specific *CXCL10* mRNA was a novel and useful target in the diagnosis of *M.tb* infection.

## Introduction

Tuberculosis (TB) is an infectious disease that seriously threatens human health, which caused 9.9 million new cases and 1.5 million deaths worldwide in 2020 (World Health Organization [WHO], 2021). Furthermore, one-fourth of the world’s population has been infected with *Mycobacterium tuberculosis* (*M.tb*) (Cohen et al., 2019). The global goal of “End TB” would be difficult to achieve due to the high burden of active TB and latent TB infection, unless there are innovations in diagnosis, treatment, and prevention methods.

The World Health Organization has recommended preventive treatment among latent TB infection population to reduce the incidence of active TB (World Health Organization [WHO], 2020). Until now, *M.tb* infection is mainly defined by tuberculin skin test (TST) or interferon-gamma release assays (IGRAs; Diel et al., 2010, 2011; Gao et al., 2015; Getahun et al., 2015). Due to the lower sensitivity of conventional *M.tb* culture or smear, as well as the lack of sputum to do the microbiological or rapid molecular tests in some suspected TB patients (World Health Organization [WHO], 2021), IGRAs have also been becoming an auxiliary method for the diagnosis of active TB. However, the TST is an *in vivo* test, and the specificity of TST is decreased seriously in BCG-vaccinated population (Pai et al., 2008). The diagnostic performance of IGRAs was decreased in immunosuppression patients, such as HIV co-infection, leukopenia, or corticosteroid and immunosuppressant drug treatment, with lower sensitivity and higher indeterminate rate (Cattamanchi et al., 2011; Jung et al., 2012; Pan et al., 2015). Furthermore, due to the longer time of antigen stimulation (18–24 h), the results of IGRA tests will not be available until the next day. In addition, ELISPOT-based IGRAs, such as T-SPOT.TB test, were mostly performed manually, which requires technicians with higher technique and may result in operation bias. Therefore, novel rapid and accurate test for diagnosis of *M.tb* infection should be developed.

Previous studies have identified an alternative biomarker in peripheral blood for *M.tb* infection, namely, interferon-gamma-induced protein 10 (IP-10) (Lu et al., 2011; Ruhwald et al., 2011, 2012; Petrone et al., 2018). IP-10 is a chemokine in CXC family, which can be expressed in a significantly higher level than conventional IFN-γ after *M.tb* antigen stimulation. Recently, IP-10 has been becoming a novel promising biomarker for TB diagnosis. Meta-analyses showed that IP-10 presented a higher sensitivity of 86% (95% CI = 80–90%) and a specificity of 88% (95% CI = 82–92%) in the diagnosis of *M.tb* infection, respectively (Qiu et al., 2019). However, most of the studies detected the expression level of IP-10 protein by ELISA or other protein examination methods, and whether *CXCL10* mRNA (the gene that transcribes for the IP-10 protein) quantification can be used in the diagnosis of *M.tb* infection was not yet elucidated. It is reported that the expression of *CXCL10* mRNA can be upregulated by about 100 times, within 2.5–8 h of *M.tb*-specific antigen stimulation (Blauenfeldt et al., 2014). Therefore, *CXCL10* mRNA as target may enable higher sensitivity in identifying individuals with *M.tb* infection (Aabye et al., 2010; Syed Ahamed Kabeer et al., 2010; Kabeer et al., 2011).

The present study aims to evaluate the diagnostic performance of the novel *M.tb*-specific *CXCL10* mRNA release assay for *M.tb* infection and analyze in accordance with the novel *CXCL10* mRNA release assay and the widely used T-SPOT.TB assay.

## Materials and Methods

### Ethical Approval

This study was performed in accordance with the guidelines of the Helsinki Declaration and was approved by the Ethics Committee of the Beijing Chest Hospital, Capital Medical University (Ethical approval number: BJXK-2017-40-01). A written informed consent was obtained from each participant before blood collection.

### Study Design and Participants

This multicenter, prospective study was performed in three hospitals in China, including the Beijing Chest Hospital (North), the Third People’s Hospital of Shenzhen (South), and the Henan Provincial Infectious Disease Hospital (Middle). Between March 2018 and November 2019, patients with suspected TB were prospectively enrolled in these three hospitals. Meanwhile, healthy controls were also enrolled. All the participants were tested with *CXCL10* mRNA release assay and T-SPOT.TB assay. The laboratory technicians were blinded to the diagnosis throughout the study, and the clinicians were blinded to the *CXCL10* mRNA release assay and T-SPOT.TB results before the end of the clinical trial enrollment. Furthermore, the laboratory technician who performed the *CXCL10* mRNA release assay was blind to the technician who performed the T-SPOT.TB. Thus, laboratory interpretation and clinical diagnosis were independent.

### Categorization of Participants

The final diagnosis was based on clinical manifestation, biochemical examinations, and histopathological, radiological, and microbiological information. All subjects were followed up for at least 6 months and were monitored for changes in their diagnostic categorization. Finally, patients were categorized as (1) Definite TB: Patients who have positive *M.tb* culture, positive Xpert MTB/RIF, positive microscopy, or positive histology were defined as definite TB. (2) Probable TB: Patients who have clinical and radiological findings of TB, and presenting well response to anti-TB treatment, but lacking microbiological or histopathological evidence of *M.tb* infection, were defined as probable TB. (3) Non-TB patients: Patients who were initially suspected of active TB, but ended up not having active TB, due to other diagnoses made, or clinical improvement occurred without recent anti-TB therapy, were defined as non-TB patients. (4) Healthy controls: Individuals who have no clinical symptoms and radiological findings of TB, and with negative TST results, were defined as healthy controls.

### Blood Collection

A total of 10 ml of peripheral blood was collected in heparin-containing vacutainer tubes from each participant, and *CXCL10* mRNA Release Assay and T-SPOT.TB Test were further performed.

### *CXCL10* mRNA Release Assay

The commercial *CXCL10* mRNA Release Assay, including the kit for blood incubation and RNA extraction (CLR001A48, InnowaveDx Co., Ltd., Suzhou, China) and kit for reverse transcription and further qPCR Test (CLR002A48, InnowaveDx Co., Ltd., Suzhou, China), were performed according to the instructions of the manufacturer. Briefly, the blood was divided into three special tubes (1.5 ml per tube): one coated with novel *M.tb-*specific peptides (peptides of ESAT6, CFP-10, and PPE68), one coated with phytohemagglutinins (PHA) as a positive control, and one without antigen coating as a negative control (Nil). The tubes were incubated immediately for 4 h at 37°C. Next, RNA was automatically extracted from the incubated whole blood (100 μl). A total of 10 μl of RNA and 10 μl of reverse transcription solution (including buffer and reverse transcriptase) were mixed and reverse transcribed to cDNA with the following procedure: 50°C for 20 min, 85°C for 2 min. RNA extraction and reverse transcription were performed using the automatic nucleic acid extraction and detection system Innovo-100 (InnowaveDx Co., Ltd., Suzhou, China). All the commercial reagents and kits for RNA extraction and reverse transcription were matched for the instrument. A total of 10 μl of cDNA was then mixed with 15 μl of quantitative real-time PCR (qPCR) mix (including enzyme, buffer, and probe). qPCR was performed on the ABI 7500 Real-time PCR System (Applied Biosystems, Inc., Foster City, CA, United States), with the following procedure: 50°C for 2 min, 95°C for 2 min, and then 40 cycles of 95°C for 10 s and 60°C for 30 s. The cycle threshold (CT) for target gene (*CXCL10*, Gene ID: 3627) and housekeeping gene (CHMP2A, Gene ID: 27243) detector was automatically determined. ΔCT (the CT value for target gene, subtract the CT value for housekeeping gene) was calculated, and this statistic was used to determine relative gene expression (Schmittgen and Livak, 2008). The relative amount of *CXCL10* mRNA in each tube with or without antigens was calculated separately. The test results were determined as indeterminate, negative, or positive according to the criteria (Supplementary Table 1).

### T-SPOT.TB Test

The T-SPOT.TB test (Oxford Immunotec Ltd., Abingdon, United Kingdom) was performed in accordance with the instructions of the manufacturer. Briefly, peripheral blood mononuclear cells (PBMCs) were isolated, and then 2.5 × 10^5^ PBMCs per well were incubated with AIMV medium (Nil control), PHA antigen (positive control), and two *M.tb*-specific antigens (ESAT-6 and CFP-10). The procedure was performed in the plates precoated with anti-interferon-γ antibodies at 37°C for 16 to 20 h. After application of alkaline phosphatase-conjugated second antibody and chromogenic substrate, the number of spot-forming cells (SFCs) in each well was automatically counted with a CTL ELISPOT system (CTL-ImmunoSpot S5 Versa Analyzer, United States). The test results were determined as indeterminate, negative, or positive according to the criteria (Supplementary Table 1).

### Statistical Analysis

Data analysis was performed using SPSS for Windows, version 21 (SPSS, Inc.). Categorical variables were compared by Pearson’s Chi-square test or Fisher’s exact test, while continuous variables were compared by Student’s *t*-test or Mann–Whitney *U*-test, as appropriate. Sensitivity and specificity were calculated to evaluate diagnostic performance for the *CXCL10* mRNA release assay and T-SPOT.TB assay. Concordance between *CXCL10* mRNA release assay and T-SPOT.TB assay was calculated using Cohen’s kappa test. The expression levels of *CXCL10* mRNA and the number of SFCs in T-SPOT.TB test were converted to lg values, in order to calculate the correlation between these two tests by Pearson’s correlation test. The criterion for statistical significance was *p*<0.05.

## Results

### Clinical Characteristics of Participants

**FIGURE 1:**
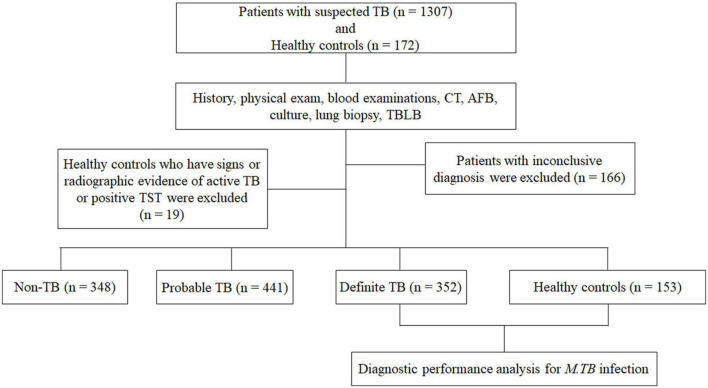
Flow chart of the study participants. Of the 1,307 patients with suspected TB and 172 healthy controls recruited, 352 definite TB patients and 153 healthy controls with lower risk of *Mycobacterium tuberculosis* (*M.tb*) infection were eligible for inclusion in the final analysis. TB, tuberculosis; CT, computed tomography; AFB, acid-fast bacilli; TBLB, transbronchial lung biopsy.

As shown in Figure 1, a total of 1,307 patients with suspected TB were enrolled from the three hospitals. According to the final diagnosis and a follow up of at least 6 months, 352 definite TB patients were included for analysis as definite *M.tb* infection status. A total of 441 probable TB patients, 348 non-TB patients, and 166 patients with inconclusive diagnosis were excluded because there were no definite *M.tb* infection evidence. Furthermore, of 172 healthy controls enrolled, only 153 persons with negative TST results and without radiological findings were included in the analysis. A total of 14 patients with definite TB were HIV positive, and there was no HIV infection among healthy controls. The detailed demographic and clinical characteristics of the participants are shown in Table 1.

**TABLE 1:** The demographic and clinical characteristics of subjects (*n* = 505).

	Definite TB	Healthy controls
Number of patients	352	153
Age (years, range)	38 (18–88)	18 (18–22)
Gender		
Male	226	79
Female	126	74
Pulmonary TB	329	–
Extrapulmonary TB	23	–
Skeleton	17	–
Lymph nodes	2	–
Urinary and genital organs	2	–
Abdomen	1	–
Meninges	1	–
HIV infection	14	0
		

### Indeterminate Results in *CXCL10* mRNA Release Assay and T-SPOT.TB Test

Among the 505 participants in the final analysis, 2 participants (0.39%) presented indeterminate result in *CXCL10* mRNA release assay, due to invalid ΔCT in the mitogen tube (>-1.2), while 7 participants (1.39%) presented an indeterminate result in T-SPOT.TB, due to higher SFCs (>10 spots) in Nil control well or lower SFCs (<20 spots) in positive control well. The proportion of indeterminate results of T-SPOT.TB was higher than that of *CXCL10* mRNA release assay, although the difference did not reach statistical significance (*p* = 0.094). HIV co-infection rate is significantly higher in participants with indeterminate results of *CXCL10* mRNA release assay and T-SPOT.TB test (33.3%, 3/9) than that in participants with valid results of *CXCL10* mRNA release assay and T-SPOT.TB test (2.22%, 11/496) (*p* = 0.0013).

### Concordance Between *CXCL10* mRNA Release Assay and T-SPOT.TB Test

Concordance between *CXCL10* mRNA release assay and T-SPOT.TB test was also analyzed in 496 participants with both valid *CXCL10* mRNA release assay and T-SPOT.TB results (Table 2). Both *CXCL10* mRNA release assay and T-SPOT.TB were positive in 312 participants and negative in 159 participants, with a good concordance (Kappa value = 0.89, *p* < 0.001). The overall concordance between the two tests was 95.0% (95% CI = 93.0–96.9%), the positive agreement was 96.3% (95% CI = 94.2–98.3%), and the negative agreement was 92.4% (95% CI = 88.5–96.4%), respectively.

**TABLE 2:** Concordance analysis between the *CXCL10* mRNA release assay and T-SPOT.TB assay.

		T-SPOT.TB		Agreement (95% CI)	Kappa value (95% CI)
		Positive	Negative		
*CXCL10* mRNA release assay	Positive	312	13	95.0 (93.0–96.9)	0.89 (0.84–0.93)
	Negative	12	159		

**FIGURE 2:**
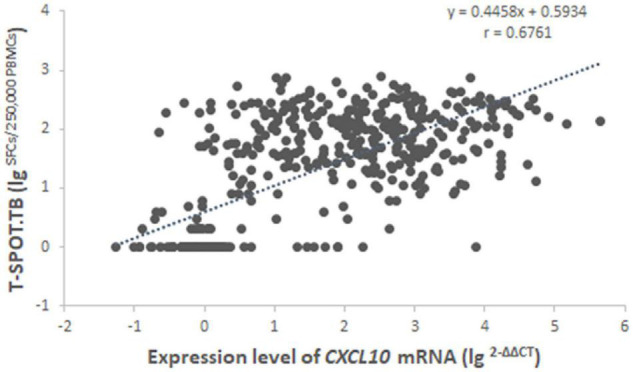
Correlation analysis between the expression level of *CXCL10* mRNA in the *CXCL10* mRNA release assay and the number of SFCs in the T-SPOT.TB assay among 496 subjects with both valid results. Regression analysis was demonstrated by linear correlation (r). SFCs, spot-forming cells.

We further investigated the association between the number of SFCs in the T-SPOT.TB test and the relative amounts of *CXCL10* mRNA in the *CXCL10* mRNA release assay (Figure 2). In most cases, the relative amount of *CXCL10* mRNA was increased when the number of SFCs in the T-SPOT.TB test was increased, indicating a moderate correlation between the expression level of *CXCL10* mRNA and the IFN-γ release in the two tests (*r* = 0.6761, *p* < 0.0001).

### Diagnostic Performance of the *CXCL10* mRNA Release Assay and T-SPOT.TB Assay for *M.tb* Infection

We evaluated the diagnostic performance of *CXCL10* mRNA release assay for *M.tb* infection based on the patients with definite TB and healthy controls (Table 3). The diagnostic sensitivity and specificity of *CXCL10* mRNA release assay was 93.9% (95% CI = 90.8–96.2%) and 98.0% (95% CI = 94.3–99.6%), respectively, while the diagnostic sensitivity and specificity of T-SPOT.TB assay were 94.5% (95% CI = 91.5–96.6%) and 100% (95% CI = 97.6–100.0%), respectively. No significant differences were detected in the sensitivity (*p* = 0.745) and specificity (*p* = 0.247) between *CXCL10* mRNA release assay and T-SPOT.TB assay.

**TABLE 3:** Diagnostic performance of the *CXCL10* mRNA release assay and T-SPOT.TB assay for *Mycobacterium tuberculosis* (*M.tb*) infection.

	Sensitivity% (95% CI)	Specificity% (95% CI)	NPV% (95% CI)	PPV% (95%CI)	LR + (95% CI)	LR- (95% CI)
*CXCL10* mRNA release assay	93.9 (90.8–96.2)	98.0 (94.3–99.6)	87.6 (82.4–91.5)	99.1 (97.2–99.7)	47.6 (15.5–145.9)	0.06 (0.04–0.09)
T-SPOT.TB	94.5 (91.5–96.6)	100.0 (97.6–100.0)	88.9 (83.8–92.5)	100.0 (100.0–100.0)	/^†^	0.05 (0.06–0.09)
*CXCL10* or T-SPOT.TB*	97.4 (95.1–98.8)	98.0 (94.3–99.6)	94.3 (89.7–96.9)	99.1 (97.3–99.7)	49.3 (16.1–151.3)	0.03 (0.01–0.05)

*NPV, negative predictive value; PPV, positive predictive value; LR+, likelihood ratio for positive test; LR−, likelihood ratio for negative value.*

*The positive result was assumed when either test was positive, and a negative result was assumed when both tests were negative.

*^†^The LR + was unavailable when the diagnostic specificity was 100%.*

The diagnostic performance of the combination of *CXCL10* mRNA release assay and T-SPOT.TB assays was also evaluated, and the positive result was assumed when either test was positive, and a negative result was assumed when both tests were negative (Table 3). Based on this standard, the diagnostic performance for *M.tb* infection was enhanced, with a better NPV value [94.3% (89.7–96.9%)].

### The Positive Rate of *CXCL10* mRNA Release Assay and T-SPOT.TB Assay Among Tuberculosis Patients With HIV Co-infection

Both the *CXCL10* mRNA release assay and the T-SPOT.TB assay are based on the host immune response to *M.tb-*specific antigens, and previous study identified HIV co-infection as the risk factor for false-negative and indeterminate IGRA results. Therefore, we further compared the diagnostic performance of *CXCL10* mRNA release assay and T-SPOT.TB assay among TB patients with HIV co-infection (Table 4). Among the 14 TB patients with HIV co-infection, all presented valid *CXCL10* mRNA release assay results, while 3 patients presented indeterminate results in T-SPOT.TB test. The positive rate of *CXCL10* mRNA release assay and T-SPOT.TB test were 92.9% (95% CI = 66.1–99.8%) and 61.5% (95% CI = 31.6–86.1%), respectively, with a significant difference in sensitivity between these two tests (*p* = 0.029). These results indicated a promising method for identifying *M.tb* infection among HIV co-infection population, although our sample size was not large enough.

**TABLE 4:** Performance of the *CXCL10* mRNA release assay and T-SPOT.TB assay among tuberculosis (TB patients) with HIV co-infection.

	Positive rate% (95% CI)	*p*-Value
*CXCL10* mRNA release assay	92.9 (66.1–99.8)	0.029
T-SPOT.TB	61.5 (31.6–86.1)	
		

## Discussion

In the present study, the concordance between the novel *CXCL10* mRNA release assay and the traditional T-SPOT.TB test was good, and the sensitivity and specificity were similar between these two tests, respectively. These results suggested that the diagnostic performance of the novel *CXCL10* mRNA release assay was consistent with the T-SPOT.TB test, indicating that *CXCL10* mRNA release assay was an alternative test for the diagnosis of *M.tb* infection. The similar performance in *M.tb* infection diagnosis may be related to the fact that the two targets belong to the same immune response signal pathway activated by *M.tb*-specific antigens. IP-10 is mainly released from the monocytes and T lymphocytes and is an IFN-γ inducible chemokine. It was reported that free IFN-γ can activate STAT1 and then amplifies JAK/STAT1 signal to release a large amount of IP-10 (Tsuboi et al., 2011). Previous studies have detected that the expression level of *IFNG* mRNA was only elevated slightly after *M.tb*-specific antigen stimulation and sustained in shorter time duration, which makes the *IFNG* mRNA not the best choice for diagnosis of *M.tb* infection (Kim S. et al., 2013; Savolainen et al., 2016). Gene expression profile identified the mRNA of multiple cytokines, such as *CXCL10*, which can be used as the detection target of the specific T-cell-based immune response to *M.tb* (Kim H. J. et al., 2013). In comparison with *IFNG* mRNA, the expression level of *CXCL10* mRNA was significantly expressed at 100-fold after *M.tb*-specific antigen stimulation, and the higher level of *CXCL10* mRNA could be sustained for a long time (Blauenfeldt et al., 2014). Thus, it is possible to explore a novel T cell based on immune response test targeting the *CXCL10* mRNA.

The diagnostic sensitivity of *CXCL10* mRNA release assay in our study was slightly higher than that in the previous Meta-analysis study [85% (95% CI = 80–88%)] (Qiu et al., 2019). This may be caused by the fact that the highly expressed *CXCL10* mRNA was used as the target. Recently, Ruhwald et al. have provided proof of high technical performance of a molecular assay detecting *CXCL10* mRNA expression, although it was reported that the *CXCL10* mRNA after 8 h of stimulation with *M.tb*-specific antigens in the QFT-IT test showed lower sensitivity than that of IP-10 protein and IFN-γ protein in the diagnosis of *M.tb* infection (Blauenfeldt et al., 2020), which was inconsistent with our results. This may be caused by the fact that the antigens used in our study were modified and could trigger stronger immune response than that in the QFT-IT test, and the appropriate stimulation time was extremely important for the utility of *CXCL10* mRNA as a target.

Interferon-gamma release assay test mainly relies on the ability of host immune response to *M.tb* antigen stimulation. It was reported that IGRA presented lower diagnostic performance in the diagnosis of TB patients with immunosuppression status, such as HIV co-infection (Cho et al., 2012; Santin et al., 2012). HIV patients were easily co-infected with *M.tb* and progressed to active TB patients (Mhango et al., 2021). The risk of HIV and *M.tb* co-infection individuals developing active tuberculosis is 20–30 times than those infected with *M.tb* alone, and the mortality of TB/HIV patients is also higher than that of patients with TB (Pawlowski et al., 2012). Therefore, the development of novel technologies that can increase the positive rate of TB with HIV infection is of great significance for timely identification and intervention of such population. In this study, we analyzed the performance of these two tests in TB patients with HIV co-infection and found that the proportion of indeterminate results of *CXCL10* mRNA release assay was lower than that of T-SPOT.TB test. Furthermore, the positive rate of *CXCL10* mRNA release assay was significantly higher than that of T-SPOT.TB test among TB patients with HIV co-infection, indicating that the detection of *CXCL10* mRNA may be more suitable than that of T-SPOT.TB for the diagnosis of *M.tb* infection in HIV co-infection patients. The increase in positive rate of *CXCL10* mRNA release assay in HIV co-infection patients may be related to the high expression level of *CXCL10* that was caused by the cascade amplification in signal pathway, and maybe there are other signal pathways involved in the release of *CXCL10* but independent of IFN-γ signal pathway or CD4^+^ T cells.

*CXCL10* mRNA release assay also has some operational advantages. Since the high expression level of *CXCL10* mRNA, the antigen stimulation time was shortened to only 4 h, which saves at least 12 h compared with traditional IGRA test. Therefore, the test result can be issued on the day of blood collection, which leads to better clinical applicability. Furthermore, automated instruments are used throughout the test, and the results are automatically interpreted, which can minimize errors or bias caused by manual operation. These advantages bring more convenience for the clinical application of *CXCL10* mRNA release assay in the diagnosis of TB.

To our knowledge, our study is the largest investigation that prospectively evaluates the diagnostic performance of *CXCL10* mRNA release assay in the diagnosis of *M.tb* infection. However, there were still some limitations in our study. The sample size of TB patients with HIV co-infection was not large enough; thus, the power for evaluating the performance of *CXCL10* mRNA release assay in this population was decreased, and further validation is needed in a larger sample size of HIV co-infected population. Furthermore, no other test was simultaneously performed as a third judge method to validate the inconsistent results between *CXCL10* mRNA release assay and T-SPOT.TB test. In addition, this study did not deeply analyze the diagnostic performance of *CXCL10* mRNA release assay in other special populations, such as patients treated with immunosuppressants, patients with other complications, and children. Further validation in the abovementioned populations should be conducted in the future.

In conclusion, the present study parallelly compared the performance of novel *CXCL10* mRNA release assay and T-SPOT.TB test in the diagnosis of *M.tb* infection. The results suggested that the overall diagnostic performance of the *CXCL10* mRNA release assay was consistent with the T-SPOT.TB test, and it presented a better positive rate in HIV co-infection patients.

## Data Availability Statement

The raw data supporting the conclusions of this article will be made available by the authors, without undue reservation.

## Ethics Statement

The studies involving human participants were reviewed and approved by the Ethics Committee of Beijing Chest Hospital, Capital Medical University. The patients/participants provided their written informed consent to participate in this study.

## Author Contributions

LP, MG, and ZZ designed the experiments. HJ, RW, QS, PR, AX, QC, XL, BD, and TC conducted the experiments. MH, GD, YC, MZ, XL, MF, and MG enrolled the subjects. LP and MH analyzed the data. LP wrote the manuscript. All authors contributed to the article and approved the submitted version.

## Conflict of Interest

The authors declare that the research was conducted in the absence of any commercial or financial relationships that could be construed as a potential conflict of interest.

## Publisher’s Note

All claims expressed in this article are solely those of the authors and do not necessarily represent those of their affiliated organizations, or those of the publisher, the editors and the reviewers. Any product that may be evaluated in this article, or claim that may be made by its manufacturer, is not guaranteed or endorsed by the publisher.
