# Supplementary material for: Diagnostic Performance of a Novel CXCL10 mRNA Release Assay for Mycobacterium tuberculosis Infection
Source: Front Microbiol. 2022 Mar 30;13:825413. doi: 10.3389/fmicb.2022.825413 (PMC9005954; doi:10.3389/fmicb.2022.825413)
Supplement: Supplementary file 1 [file Table_1.DOCX]

**Supplementary table 1. The results interpretation criteria of *CXCL10* mRNA release assay and T-SPOT.TB assay.**

| **Interpretation** | **Nil^*^** | **TB antigen response^†^** | **PHA response^§^** |
| --- | --- | --- | --- |
| ***CXCL10* mRNA release assay** | | | |
| Positive ^**^ | Any | ≤-1.04 | Any |
| Negative **^††^** | Any | >-1.04 | ≤-1.2 |
| Indeterminate **^§§^** | Any | >-1.04 | >-1.2 |
| **T-SPOT.TB assay** | | | |
| Positive ^**^ | ≤10 | ≥6 | Any |
| Negative **^††^** | ≤10 | <6 | ≥20 |
| Indeterminate **^§§^** | ≤10 | <6 | <20 |
|  | >10 | Any | Any |

**^*^** The amount of IFN-γ or *CXCL10* mRNA in tube or well without antigen.

**^†^** The amount of IFN-γ or *CXCL10* mRNA in *M.tb* antigen tube or well minus that in Nil control tube or well.

**^§^** The amount of IFN-γ or *CXCL10* mRNA in PHA antigen tube or well minus that in Nil control tube or well.

^**^ Interpretation indicating that *M.tb* infection is likely.

**^††^** Interpretation indicating that *M.tb* infection is not likely.

**^§§^** Interpretation indicating an uncertain likelihood of *M.tb* infection.
